# Analysis of Mechanisms of T-2 Toxin Toxicity Using Yeast DNA Microarrays

**DOI:** 10.3390/ijms9122585

**Published:** 2008-12-11

**Authors:** Yumiko Iwahashi, Emiko Kitagawa, Hitoshi Iwahashi

**Affiliations:** 1 National Food Research Institute, 2-1-12 Kannondai, Tsukuba-shi, Ibaraki 305-8642, Japan; 2 Health Technology Research Center, National Institute of Advanced Industrial Science and Technology, Osaka, Japan. E-Mails: emiko.kitagawa@roche.com (E. K.); hitoshi.iwahashi@aist.go.jp (H. I.)

**Keywords:** T-2 toxin, DNA microarray, yeast, mycotoxin

## Abstract

T-2 toxin is a mycotoxin that belongs to a group of type A tricothecenes found in agricultural products. The cytotoxicity of T-2 toxin was characterized by analysis of the yeast transcriptome upon challenge with T-2 toxin. Interestingly, T-2 toxin-induced yeast gene expression profiles were found to be similar to profiles obtained following cycloheximide treatment. Moreover, T-2 toxin treatment was found to activate facilitators, gluconeogenesis and cell arrest related genes such as mitogen-activated protein kinase genes (FUS3). T-2 toxin attacks the membrane and as a result the membrane transport system was disturbed. A large number of genes are induced to restore the toxicity caused by T-2 toxin. However, the data did not suggest that DNA damage by alkylation (Mag1, a gene 3-methyl-adenine DNA glycosylase, 0.46-fold down regulated), no induction of DNA repair mechanisms such as recombination (RAD26, RAD52 and etc.) and excision repair (RAD7, RAD14, RAD16, RAD23 and etc.). These results suggested that the toxicity of the T-2 toxin was due to the disturbance of the cell membrane of the yeast cell and that T-2 toxin caused mild mutagenesis.

## 1. Introduction

T-2 toxin is a mycotoxin that belongs to a group of typeA trichothecenes produced by several fungal genera, including *Fusarium* species. *Fusarium sporotrichioides* and *F. poae* are contaminants of certain agricultural commodities and are also species of economic importance capable of producing the potent trichothecene T-2 toxin. T-2 toxin has been found as a contaminant in cereals, including corn, oats and wheat. This toxin has been shown to cause a variety of toxic effects in both experimental animals and humans [1]. It induces apoptosis in the liver, placenta and fetal liver in pregnant rats [2]. Among the trichothecenes, T-2 toxin has the greatest cytotoxicity. Lymphocytes are more sensitive to T-2 toxin than other cultured cell lines and this corresponds well with data from *in vivo* experiments showing that trichothecenes act as immunosuppressive agents [3]. Specifically, T-2 toxin effects on human lymphocytes include blunting of mitogen-induced blast transformation, inhibition of antibody-dependent cellular cytotoxicity, and suppression of natural killer activity [4].

Recent DNA microarray technologies have been developed which allow for the simultaneous detection of the expression of many genes. In the current experiment, we used yeast as the model eukaryotic cell because its complete genomic information is available and it is very easy to use. The application of this technology to the field of toxicology has been demonstrated. For example, patulin-induced yeast gene expression profiles were found to be similar to gene expression patterns obtained after treatment with the antifungal chemicals thiuram, maneb and zineb. Moreover, patulin treatment was found to activate protein degradation (particularly proteasome mediated degradation) sulfur amino acid metabolism, and the oxidative stress defense system [5]. In addition, we studied the toxicity of citrine to yeast cells using the ORF DNA microarray system and Oligonucleotide DNA microarray systems. Both DNA microarray results suggested that the oxidative stress was main toxicity but this stress did not lead to DNA damages. This observation was different from toxicity of another mycotoxins of patulin to yeast cells [6].

In the present study, we have examined the detailed gene expression changes in yeast exposed to T-2 toxin. T-2 toxin. The cell membrane of yeast was perturbated, and/or influenced and caused the cell arrest by T-2 toxin treatment. And more it was thought that the mutagenesity was low because the T-2 toxin hardly influenced the restoration enzyme genes.

These results suggested the possibility to use the yeast transcriptome system for the evaluation of natural chemical that are difficult to get by organic synthesis. The first screening method of the toxicity of these organic matters is developed, and the thing to decrease the animal experiment is the final purpose.

## 2. Results and Discussion

### 2.1. Conditions for T-2 toxin treatment

**Figure 1.**
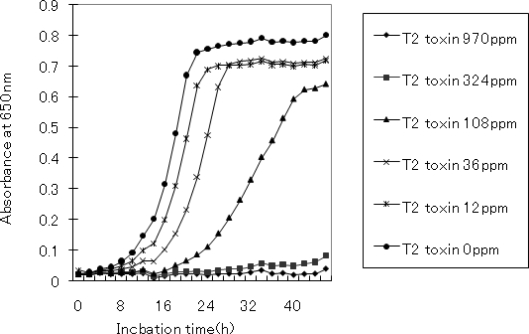
Effect of T-2 toxin on yeast growth. Varying amounts of T-2 toxin, dissolved in DMSO at concentration of 2,000 ppm, were added to YPD medium at the indicated concentration.

Initially, we characterized the effect of T-2 toxin treatment on yeast growth. Biological and physiological characterization of the effects of T-2 toxin treatment was necessary to ensure that the induction or repression of specific genes is due to treatment effects. Lack of growth inhibition would merely show that the condition studied did not cause sufficient cellular stresses and that the results obtained may not necessarily reflect the full stress response. Figure 1 shows yeast growth as a function of T-2 toxin concentrations. No growth was observed at concentrations greater than 324 ppm while inhibition could be seen at concentrations greater than 12 ppm. Based on this dose-response analysis, 108 ppm T-2 toxin was found to inhibit growth in a non-lethal manner, and therefore chosen as the test concentration in our experiments.

### 2.2. Overview of T-2 toxin induced genes, cellular location and functional distribution

Among the 6,131 ORFs that exhibited intensities over the cut-off value with p-values less than 0.05, 515 genes exhibited greater than 2-fold higher intensities and 490 genes had less than 0.5-fold intensities following T-2 toxin treatment. The highly induced genes are listed in Table 1. Among the 45 genes induced more than 5-fold, 11 are transporter genes. Plasma membrane transporters mediate extrusion of intracellular compounds into the medium, while others, located in intracellular membranes, catalyze efflux from or within the mitochondria, vacuole, peroxisomes or secretion organelles. These membrane proteins are generally classified into three main categories: channels, facilitators (also named transporters, permieases, or carriers) and pumps (ATPases). Among the genes induced more than 5-fold by T-2 toxin treatment, all belonged to the facilitator subcategory. A large number of facilitators have been induced by T-2 toxin, indicating that T-2 toxin has a large influence on yeast plasma membranes, and that the movement of the materials is disrupted. In addition, 32.7% of the altered genes belonged to the nucleus and mitochondria. For instance, Cat2 (YML042W, 5.7-fold induction), carnitine acetyl-CoA transferase, is present in mitochondria and peroxisomes, and transfers activated acetyl groups to carnitine to form acetylcarnitine, which can be shuttled across membranes. Crc1 (Yor100C, 7.5-fold induction) is a mitochondrial inner membrane carnitine transporter, required for carnitine-dependent transport of acetyl-CoA from peroxisomes to mitochondria during fatty acid beta-oxidation. Crc1 is located in the mitochondria and belongs to the mitochondrial carrier (MCF) family. A fatty liver was observed in experiments utilizing rats, suggesting that the fatty change in the rat liver may be related to oxidative stress caused by T-2 toxin [7]. T-2 toxin may cause lipid peroxidation and induce mitochondrial dysfunction. Moreover, it is understood that T-toxin causes cell cycle arrest with pheromone receptivity and induction of MAPK signaling pathway genes such as Ste2, Fus3, and Fal1.

**Figure 2.**
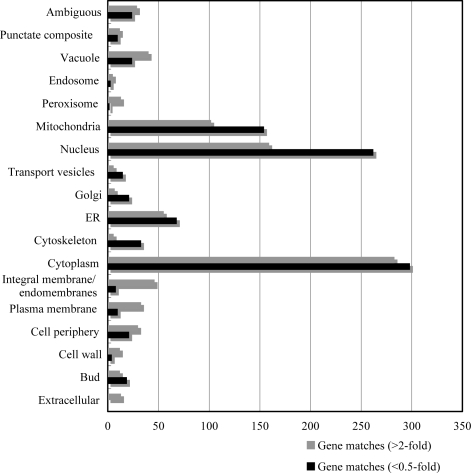
Locational distribution of highly induced genes by T-2 toxin treatment.

The 515 induced and 490 reduced genes were analyzed for their vocational distribution using MIPS functional categories (Figure 2). Cellular localization in the group induced by 2-fold or more feature members of the plasma membrane and integral membrane groups, etc., as well as members of the nuclear and mitochondrial groups. Consistent with this, T-2 toxin treatment inflicted abundant damage to the yeast cell membranes, and it is therefore suggested to cause nuclear and mitochondrial dysfunction.

**Figure 3.**
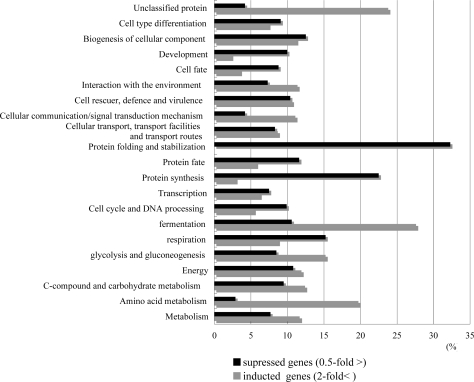
Functional categories of induced or suppressed genes by T-2 toxin.

The altered genes were classified using the MIPS functional categories (Figure 3). Many of the induced genes belonged to the following categories: “metabolism, 11.7%”, “energy, 11.9%”, “cell rescue, 11.4%”, and “interaction with the cellular environment, 11.4%”. Conversely, many of the reduced genes belonged to the following categories: “energy, 10.8%”, “protein synthesis, 22.6%”, “protein fate, 11.6%”, “protein with binding function or cofactor requirement, 10.7%”, “cell rescue, defense and virulence, 10.5 %”, and “development, 10.1 %”.

### 2.3. T-2 toxin causes the oxidative stress, and causes the energy scarcity in yeast

In Table 2, subcategories in metabolism and energy are listed with the number of induced or suppressed genes.

Among the 177 metabolism genes, 48 belong to amino acid metabolism (mostly related to assimilation of ammonia) including 17 related to metabolism of the glutamate group, 10 related to metabolism of arginine, 13 related to metabolism of the aspartate family and 15 related to metabolism of the cysteine-aromatic group. The enzymes of glutathione synthesis pathway that take part in the detoxification of the heavy metals were not induced though the glutamate group was induced (data not shown). It is thought that glutathione does not take part in the detoxication of T-2 toxin.

Furthermore, there were many *C*-compound and carbohydrate metabolism related genes induced by more than 2-fold by T-2 toxin treatment, and 23.8 % of these genes were related to stress or detoxification. In this subcategory, Fdh1 (6.6-fold induction) is strongly similar to *H. polymorpha* formate dehydrogenase gene, takes part in NADH generation, and has detoxifying activity. Furthermore, it is thought that the genes Dak1, Dak2, Add4, Add6, Add14, and Add16 are genes induced by oxidative stress. Additionally, Sip4 (15.7-fold induction) encodes a zinc-cluster protein that can bind to CSRE (carbon source-responsive element) motifs during periods of glucose deficiency, and is a structural component of gluconeogenesis activated and regulated by Snf1p protein kinase [8]. The Snf1p (1.96-fold induction) kinase complex, which phosphorylates serine and threonine residues, is essential for regulating the transcriptional changes associated with glucose derepression through its activation of the transcriptional activators Cat8p and Sip4p. The Snf1/AMP-activated protein kinase (AMPK) family plays a central role in responses to metabolic stress and regulation of energy homeostasis in eukaryotes. The AMP-activated/Snf1 protein kinase subfamily members are metabolic sensors of the eukaryotic cell [9]. Snf1p is known or predicted to phosphorylate a wide range of substrates. The kinase activity of Snf1p is under multiple types of regulation. Under low-glucose conditions, the catalytic domain is bound by Snf4p, which alleviates the auto inhibition from the Snf1p regulatory domain [10–11]. Snf1p is similar to human PRKAA2, which is implicated in pancreatic carcinoma and may be an important target for drug development against diabetes, obesity, and other diseases. In mammals, AMPK regulates many metabolic processes, notably glucose and lipid metabolism, and controls transcription and protein synthesis to maintain energy balance. AMPK is activated by hormones and by stresses that deplete cellular ATP, thereby elevating the AMP: ATP ratio. In humans, it is a target of drugs used in treating type 2 diabetes [12–13]. Membrane dysfunction caused by T-2 toxin is thought to be due to energy scarcity in the yeast cell resultant from the above noted alterations in metabolic gene expression. In addition, T-2 toxin induced fructose-1, 6-bisphosphatase (2.9 fold induced) expression, a key regulatory enzyme in the gluconeogenesis pathway required for glucose metabolism [14–16]. It is thought that cellular movement of glucose is inhibited by the T-2 toxin treatment. However, enough glucose exists in the medium, and the glucose deprivation induces the pathway of gluconeogenesis-coded genes.

The genes related to the control of the electron transfer system for yeast, especially cytocrome c related genes, were suppressed by the T-2 toxin treatment. Cytochrome c oxidase catalyzes the terminal step in the electron transport chain involved in cellular respiration. This multisubunit enzyme of the mitochondrial inner membrane, also known as Complex IV, is composed of 3 core subunits encoded by the mitochondrial genome (Cox1p, Cox2p, and Cox3p) and eight additional subunits encoded by nuclear genes (Cox4p, Cox5Ap or Cox5Bp, Cox6p, Cox7p, Cox8p, Cox9p, Cox12p, and Cox13p). At least 70% of the total genes related to cytochrome c were decreased by the T-2 toxin treatment (data not shown). It is thought that this results in energy depletion in the yeast cell. The phenotype of a mutation affecting any of the genes encoding cytochrome c oxidase subunits, or any of the multiple genes required for expression or assembly of the subunits, is a decrease or block in aerobic growth. The inability to respire is not lethal since *S. cerevisiae* can grow by fermentation, but non-respiring cells grow more slowly than respiratory-competent cells, even on glucose-containing medium, resulting in smaller colony size. This provides further support for the contention that T-2 toxin induces energy scarcity in the yeast cell.

### 2.4. T-2 toxin induced cell arrest, decreased DNA repair system, and inhibited the synthesis and the processing of the ribosome

**Figure 4.**
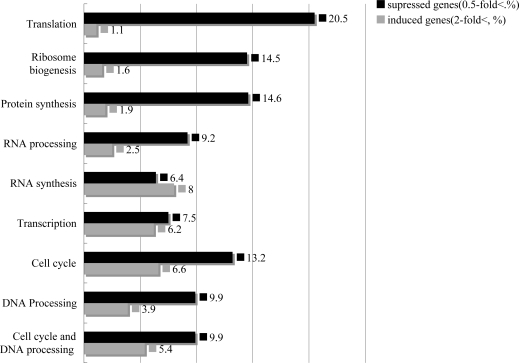
Sub-categories of cell cycle, DNA processing, transcription and protein synthesis.

We listed further the categories in cell cycle and DNA processing, transcription and protein synthesis in Figure 4. In the cell cycle and DNA processing category, 100 genes (9.9%) suppressed while 54 genes (5.4%) were induced. Moreover, in the subcategory of DNA procession and cell cycle, the suppressed genes were more than the induced genes.

Ybl016 (Fus3p, 8.4-fold induced) [17–18] and Yjl157c (Far1, 4.0-fold induced) [19] belong to the same category. Ybl016w is mitogen-activated serine/threonine protein kinase involved in mating; phosphoactivated by Ste7p [20] and Yjl157c [21] is cyclin-dependent kinase inhibitor that mediates cell cycle arrest in response to pheromones. These two genes were thought to be evidence of strong proliferation control due to cell cycle arrest induced by the toxicity of the T-2 toxin.

In the transcription and protein synthesis categories, many genes concerning RNA were down regulated, especially those concerning ribosome biogenesis and the RNA processing (particularly genes involved in processing rRNA). However, few genes were induced in the protein synthesis category, and many genes (70 genes, 20.5%) were down regulated. We thought that strong protein synthesis inhibition was caused by the T-2 toxin treatment because most of the genes were ribosome biogenesis related (Figure 4).

There were no genes induced and 30 genes were down regulated in the protein folding and stabilization subcategory. It was thought that the T-2 toxin repressed the damage recovery function because of the repressed of heat shock proteins, or proteins with similar function, by T-2 toxin treatment (accounting for 55%, data not shown). On the other hand, in the modification by phosphorylation, dephosphorylation, and autophosphorylation sub subcategories, many serine/threonine protein kinase genes (50%) were induced, and Fus3 (mitogen-activated serine/threonine protein kinase involved in mating; phosphoactivated by Ste7p; substrates include Ste12p, Far1p, Bni1p, Sst2p; inhibits invasive growth during mating by phosphorylating Tec1p, promoting its degradation) that especially caused the cell arrest was highly induced (8.4-fold). Fus3, Kss1and Slt2 play an important role in cell cycle arrest and glucose starvation, etc.

In addition, the cyclin dependent kinase inhibitor Far1p is able to inactivate Cdc28p/Clnp complexes, and thus helps stop the cell cycle at START in G1 [22]. This is important for mating, in which haploid cells of opposite mating type synchronize their cell cycles by arresting at START, so that they can fuse and become a diploid. Transcription of Fr1 is induced by the mating pheromone, in a Ste12p-dependent manner [23]. Far1p levels are also controlled by ubiquitin-mediated proteolysis; Far1p is unstable throughout the cell cycle except during G1 [24]. Phosphorylation by the MAP kinase homolog Fus3p may help regulate Far1p's association with the Cdc28p/Cln2p complex [25]. Far1p may also serve another function in the cell, as part of a Cdc24p-G/beta/gamma-Far1p complex that acts as a landmark for oriented growth during mating. This would be made possible by the movement of Far1p from the nucleus to the cytoplasm in response to the mating pheromone [26].

### 2.5. Mutagenicity in the T-2 toxin

Genes involved in the repair of double-stranded breaks in DNA are listed in Table 3. In *S. cerevisiae*, nucleotide excision repair (NER) is mediated by Rad1p, Rad2p, Rad4p, Rad7p, Rad10p, Rad14p, Rad16p, Met18p, the transcription factor TFIIH, and the heterotrimeric complex RPA (Rfa1p, Rfa2p, Rfa3p). Here, we observed that the expression of Rad51 and 57 is induced. However, it is thought that the observed level of induction of 1.5-fold was moderate, while the expression other genes included in Table 3 were largely repressed. These genes are members of the Rad52 epitasis group. These are proteins that stimulate strand exchange by facilitating Rad51p binding to single-stranded DNA, annealing complementary single-stranded DNA and are involved in the repair of double-strand breaks in DNA during vegetative growth and meiosis [27]. However, a gene that is involved in protecting DNA against alkylating agents, initiates base excision repair by removing damaged bases to create basic sites that are subsequently repaired (Mag1, a gene 3-methyl-adenine DNA glycosylase), was not induced by T-2 toxin treatment. It should be noted that other repair genes, such as for base excision repair (Ntg1, Ntg2 and Apn), mismatch repair (Msh), and translation synthesis (Rev1, Rev3 and Rev7), were not induced to significant extent. From the above-mentioned observations, it was thought that T-2 toxin treatment did not damage the DNA repair system. Therefore, it is thought that the mutagenicity of T-2 toxin was low and mechanistically different from the effects of patulin.

### 2.6. Biomarker Genes for T-2 toxin and cluster analysis

**Figure 5.**
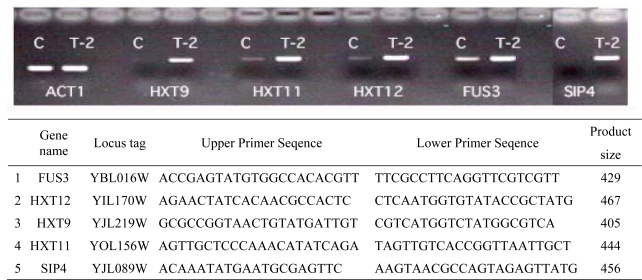
Confirmation of T-2 toxin stimulated gene induction by RT-PCR. Gene names are provided at the bottom and the primers used are shown in the box.

DNA microarray can be used to understand chemotoxicity and the selection of reporter genes indicative of such toxicity. The genes highly induced by T-2 toxin treatment are candidate reporter genes. To confirm the possibility of using these genes as reporters, we selected Hxt9, Hxt11, Hxt12, Fus3 and Sip4 (Figure 5). All genes yielded more RT-PCR product compared to the control.

To characterize the response to the T-2 toxin, cluster analysis of the mRNA expression profile [28, 29] was performed using Pearson correlation. The profile of the T-2 toxin treatment was found to cluster with that of the protein synthesis inhibitor cycloximide (data not shown).

## 3. Experimental Section

### 3.1. Strains, growth conditions and T-2 toxin treatment

*Saccharomyces cerevisiae* strain S288C (*Mat alpha SUC2 mal mel gal2 CUP1*) was grown in YPD medium (2% polypeptone, 1% yeast extract, 2% glucose) at 25 °C as a pre-culture for 2–3 days. This strain was used because the DNA microarray probes were produced using S288C DNA as the template for PCR. T-2 toxin was purchased from MP Biochemicals (Irvine, CA, USA) and was dissolved in DMSO. The stock solution was added directly to the YPD medium or the YPD medium containing yeast cells, such that it was diluted more than 100-fold. The yeast cells were grown until OD_600_ = 1 at 25 °C and collected 2 hours after the T-2 toxin was added to cell culture.

### 3.2. DNA microarray analysis

DNA microarray analysis was carried out on three independent cultures as previously described [28–32]. Total RNA was isolated by the hot-phenol method. Poly(A)^+^ RNA was purified from total RNA with Oligotex-dT30 mRNA purification kits (Takara, Kyoto, Japan). Two to four micrograms of poly(A)^+^ RNA were used for each labeling experiment, and the same amount of each poly(A)^+^ RNA was used on each slide. Messenger RNA from T-2 toxin treated cells was labeled with Cy5-dUTP, and the mRNA of reference untreated cells was labeled with Cy3-dUTP, fluorescently. Two color-labeled cDNAs were mixed and hybridized with a yeast microarray (ver.2.0, DNA Chip Research, Inc., Yokohama, Japan) for 24–36 h at 65 °C. On this microarray, ORFs of 200–8,000 bp length (0.1–0.5 ng) had been spotted, such that 6,037 genes could be analyzed under these conditions [30]. Details of the microarray procedure and validation studies with our conditions have been previously described [30.^31^]. Detected signals for each ORF were normalized by intensity dependent (LOWESS) methods [33]. Genes called as induced or repressed were those passing a one-sample t-test (p-value cut-off 0.05) as well as showing 2-fold higher or lower expression compared to the control. All the experiments were done three times independently. These selected genes were characterized for function according to the functional categories established by MIPS [34] and the SGD [35]. The data obtained in this experiment has been assigned accession number GSE10103 in the Gene Expression Omnibus Database [33].

Cluster analysis of mRNA expression profiles after T-2 toxin treatment. Hierarchical cluster analysis was performed using GeneSpring ver. 7.3.1 software (Agilent Technology) [32]. The clustering algorithm arranges conditions according to their similarity in expression profiles across all conditions, such that conditions with similar patterns are clustered together in a taxonomic tree. The setting for the calculation was as follows: Pearson Correlation measured the similarity; the separation ratio was 1.0; the minimum distance was 0.001; and 3,800 ORFs were used for the calculation. These 3,800 ORFs were selected on the basis of having previously exhibited higher than average intensities in another trial [33].

RT-PCR. Reverse transcriptase-polymerase chain reaction (RT-PCR) was carried out to select biomarker genes and to confirm the microarray results as described previously [36]. The gene names (systematic names), and forward and reverse primer sequences (5’ to 3’) are presented in Figure 2. RT-PCR was performed using the One Step RNA PCR Kit (Takara). Temperature and cycle conditions were as follows: 70 °C for 3 min, 50 °C for 30 min, 92 °C for 2 min, 20–30 cycles of (94 °C for 30 sec, 55 °C for 30 sec, 72 °C for 45 sec), and 72 °C for 10 min.

## 4. Conclusions

We characterized alterations in gene expression, using DNA microarray, to clarify the effects of T-2 toxin on yeast cells. T-2 toxin treatment induced and repressed the expression of 515 and 490 genes, respectively. The most highly induced genes (> 5-fold) belonged to the facilitator class, demonstrating that T-2 toxin had large influence on the plasma membrane of yeast, resulting in aberrant movement of the materials. As a consequence, we hypothesized that the induction of Hxt genes and gluconeogenesis related genes caused a state of glucose depletion.

Additionally, T-2 toxin inhibited ribosome function, and caused the inhibition of protein synthesis. T-2 toxin treatment also induced Fus3p (mitogen-activated serine/threonine protein kinase) and Far1 (cyclin-dependent kinase inhibitor that mediated cell cycle arrest) expression. Induction of these genes causes cell cycle arrest in yeast. DNA microarray analysis in pregnant rats suggests that the mitogen-activated protein kinase (MAPK) pathway might be involved in the mechanism of T-2 toxin-induced apoptosis [37]. The attack of the T-2 toxin to the membrane causes the transformation of the membrane, and, as a result, the flow of the material inside and outside of the cell is perturbated. And, it seems that many genes are induced to face with various troubles caused by the effect on the membrane. Therefore, toxicity by the T2 toxin cannot be explained as the result of one single effect but it is due to multiple ones. In this paper, correlation with the induced and /or repressed genes and chemical nature/structure of the T-2 toxin cannot be clarified yet.

The genes involved in DNA repair, such as Rad group, Mag1 (3-methyladenine DNA glycosidase) or MSH group (DNA mismatch repair protein), were not markedly induced. Therefore, it was proposed that T-2 toxin did not cause DNA damage, that only low levels of repair were occurring, and that there was no T-2 toxin-induced mutagenicity.

## Figures and Tables

**Table 1.** List of highly induced genes by T-2 toxin treatment in yeast.

Systematic	Normalized	Common	Description
YJL089W	15.7	SIP4	interacts with SNF1 protein kinase
YOL156W	15.7	HXT11	Hxt family protein with intrinsic hexose transport activity
YJL219W	12.8	HXT9	Hxt family protein with intrinsic hexose transport activity
YNL279W	12.4	PRM1	similarity to S. pombe coiled-coil protein of unknown function
YOL052C-A	12.0	DDR2	heat shock protein DDRA2
YHR137W	11.4	ARO9	aromatic amino acid aminotransferase II
YIL170W	10.7	HXT12	strong similarity to sugar transport proteins
YFL058W	9.4	THI5	pyrimidine biosynthesis protein
YBL016W	8.4	FUS3	mitogen-activated protein kinase (MAP kinase)
YBR296C	8.2	PHO89	Na+/phosphate co-transporter
YOR303W	7.8	CPA1	arginine-specific carbamoylphosphate synthase, small chain
YOR100C	7.5	CRC1	mitochondrial carnitine carrier
YMR017W	7.5	SPO20	Dbf2p interacting protein
YGL255W	7.4	ZRT1	zinc transporter I
YFL026W	7.3	STE2	pheromone alpha-factor receptor
YHR018C	7.3	ARG4	arginosuccinate lyase
YGR213C	7.2	RTA1	involved in 7-aminocholesterol resistance
YHL021C	6.9	FMP12	weak similarity to *Pseudomonas* gamma-butyrobetaine hydroxylase
YJR078W	6.8	BNA2	tryptophan 2,3-dioxygenase
YAR020C	6.7	PAU7	strong similarity to members of the Srp1p/Tip1p family
YJR156C	6.6	THI11	thiamine regulated gene, homologous to nmt1a in S. pombe
YOR388C	6.6	FDH1	strong similarity to H.polymorpha formate dehydrogenase
YDR380W	6.5	ARO10	similarity to Pdc6p, Thi3p and to pyruvate decarboxylases
YPL280W	6.5	HSP32	strong similarity to YMR322c and YDR533c
YOL119C	6.3	MCH4	similarity to monocarboxylate transporter proteins
YOR222W	6.3	ODC2	mitochondrial 2-oxodicarboxylate carrier
YJR109C	6.2	CPA2	arginine-specific carbamoylphosphate synthase, large chain
YPL250C	6.2	ICY2	interacting with the cytoskeleton
YJL088W	6.2	ARG3	ornithine carbamoyltransferase
YIL117C	6.2	PRM5	similarity to hypothetical protein YNL058c
YGR055W	6.0	MUP1	high affinity methionine permease
YHR021W-A	6.0	ECM12	probably involved in cell wall structure or biogenesis
YFL053W	6.0	DAK2	dihydroxyacetone kinase
YCL055W	5.9	KAR4	regulatory protein required for pheromone induction of karyogamy genes
YPL135W	5.8	ISU1	strong similarity to nitrogen fixation protein (nifU)
YLR142W	5.8	PUT1	proline oxidase
YMR096W	5.7	SNZ1	stationary phase protein
YML042W	5.7	CAT2	carnitine *O*-acetyltransferase
YPL223C	5.7	GRE1	induced by osmotic stress
YMR159C	5.7	ATG16	coiled-coil protein required for autophagy
YDL244W	5.6	THI13	strong similarity to Thi5p, YJR156c, YNL332w and A. parasiticus, *S. pombe* nmt1 protein
YGR161C	5.3	RTS3	hypothetical protein
YNL125C	5.3	ESBP6	similarity to YKL221w and human X-linked PEST-containing transporter
YKL217W	5.0	JEN1	Lactate and pyruvate permease

**Table 2.** Genes that relate to stress and detoxification among genes related to C-compound and carbohydrate metabolism, glycolysis and glyconeogenesis induced by >2-fold by T-2 toxin.

Systematic No.	Fold	Common name	Description	Function
***C*-compound and carbohydrate metabolism**				
YOR388C	6.6	FDH1	NADH regeneration	detoxification
YFL053W	6.0	DAK2	dihydroxyacetone kinase	stress
YDL243C	4.3	AAD4	strong similarity to aryl-alcohol dehydrogenase	oxidative stress
YFL056C	4.0	AAD6	strong similarity to aryl-alcohol dehydrogenases	oxidative stress
YCR105W	3.8	ADH7	NADP(H)-dependent alcohol dehydrogenase	oxidative stress
YFL014W	3.7	HSP12	heat shock protein	heat shock
YFL057C	3.1	AAD16	aryl-alcohol dehydrogenase	oxidative stress
YAL021C	3.1	CCR4	transcriptional regulator	response to drug
YOR178C	2.8	GAC1	ser/thr phosphoprotein phosphatase 1, regulatory chain	heat shock protein binding
YNL331C	2.6	AAD14	strong similarity aryl-alcohol reductase	oxidative stress
YKL062W	2.5	MSN4	transcriptional activator	oxidative stress
YDL066W	2.5	IDP1	isocitrate dehydrogenase (NADP+), mitochondrial	NADPH production
YER073W	2.4	ALD5	aldehyde dehydrogenase (NAD+), mitochondrial	oxidative stress
YML070W	2.3	DAK1	dihydroxyacetone kinase, induced in high salt	response to stress
YGR019W	2.3	UGA1	4-aminobutyrate aminotransferase	oxidative stress
**Glycolysis and glyconeogenesis**				
YJL089W	15.7	SIP4	interacts with SNF1 protein kinase	the positive regulation of gluconeogenesis
YCR105W	3.8	ADH7	NADP(H)-dependent alcohol dehydrogenase	oxidative stress
YBR105C	3.4	VID24	required for vacuolar import and degradation of Fbp1p	negative regulation of gluconeogenesis
YCL040W	2.9	GLK1	aldohexose specific glucokinase	glucose import
YLR377C	2.9	FBP1	fructose-1,6-bisphosphatase	gluconeogenesis
YOR347C	2.9	PYK2	pyruvate kinase, glucose-repressed isoform	glycolysis
YJL155C	2.1	FBP26	fructose-2,6-bisphosphatase	gluconeogenesis

**Table 3.** Genes involved in the repair of double-stranded breaks in DNA.

Systematic No.	Normalized	Common	Description
YDR076W	2.5	RAD55	DNA repair protein
YER095W	1.5	RAD51	DNA repair protein
YDR004W	1.5	RAD57	DNA repair protein
YER171W	1.3	RAD3	DNA helicase/ATPase
YER162C	1.3	RAD4	excision repair protein
YER173W	1.2	RAD24	cell cycle checkpoint protein
YER095W	1.5	RAD51	DNA repair protein
YDR004W	1.5	RAD57	DNA repair protein
YER143W	1.3	DDI1	induced in response to DNA alkylation damage
YPL164C	1.2	MLH3	insertion and deletion mismatch repair protein
YBR272C	1.1	HSM3	mismatch repair protein
YJR052W	1.1	RAD7	nucleotide excision repair protein
YCR092C	1.1	MSH3	DNA mismatch repair protein
YJR035W	1.1	RAD26	DNA repair and recombination protein
YML032C	1.0	RAD52	recombination and DNA repair protein
YGL163C	1.0	RAD54	DNA-dependent ATPase of the Snf2p family
YML095C	1.0	RAD10	DNA repair protein
YDL059C	0.9	RAD59	recombination and DNA repair protein
YPL022W	0.9	RAD1	component of the nucleotide excision repairosome
YDR030C	0.8	RAD28	protein involved in the same pathway as Rad26p
YNL250W	0.8	RAD50	DNA repair protein
YMR201C	0.8	RAD14	nucleotide excision repair protein
YGR258C	0.8	RAD2	structure-specific nuclease of the nucleotide excision repairosome
YBR114W	0.8	RAD16	nucleotide excision repair protein
YLR032W	0.7	RAD5	DNA helicase
YHR120W	0.7	MSH1	DNA mismatch repair protein, mitochondrial
YGL058W	0.6	RAD6	E2 ubiquitin-conjugating enzyme
YPL153C	0.5	RAD53	ser/thr/tyr protein kinase
YEL037C	0.5	RAD23	nucleotide excision repair protein (ubiquitin-like protein)
YER142C	0.5	MAG1	3-methyladenine DNA glycosylase
YNL082W	0.4	PMS1	DNA mismatch repair protein
YDR217C	0.4	RAD9	DNA repair checkpoint protein
YDR097C	0.4	MSH6	DNA mismatch repair protein
YOL090W	0.4	MSH2	DNA mismatch repair protein
